# Substance Use Among Iranian Youth: A Nationwide Cross‐Sectional Study on the Prevalence, Pattern, and Its Associated Factors

**DOI:** 10.1002/hsr2.71604

**Published:** 2025-12-03

**Authors:** Farshad Hadianfard, Habib Hadianfard, Alireza Sadeghi, Erfan Taherifard

**Affiliations:** ^1^ Student Research Committee, Shiraz School for Medicine Shiraz University of Medical Sciences Shiraz Iran; ^2^ Department of Clinical Psychology, School of Education and Psychology Shiraz University Shiraz Iran; ^3^ Health Policy Research Center, Shiraz School for Medicine Shiraz University of Medical Sciences Shiraz Iran

**Keywords:** adolescents, high school, Iran, students, substance use, youths

## Abstract

**Background and Aims:**

Substance use poses a considerable challenge with adverse consequences on physical and psychological well‐being, and societal dynamics, particularly among adolescents and young adults. This cross‐sectional study aimed to investigate substance use prevalence and its associated factors among high school students across Iran.

**Methods:**

Eligible participants were high school students from all grades studying in the fields of Theoretical branches. An online questionnaire comprised of two sections was distributed to the target population utilizing a convenience sampling method; the first section of the questionnaire focused on demographic data, while the second gathered ever and recent substance use history. Multivariable Poisson regression was used to identify independent factors associated with the number of different substances used by the participants.

**Results:**

A total of 9402 individuals participated in this study, comprised of 56.9% males and 43.1% females. Of these, 3687 (39.2%) reported ever using at least one substance, and 2691 (28.6%) reported recent use. Among ever‐users, 53.0% used more than one substance type; among recent users, 47.1% used more than one type. A greater number of substances used (ever or recent) was associated with older age, being married, attendance at nonpublic schools, lower grade point average, single‐parent household, and lower parental education.

**Conclusion:**

A notable percentage of high school students had the experience of ever and recent substance use, underscoring the need for further preventive measures to address substance use among Iranian youth, with a focus on specific demographics.

## 1 Introduction

Substance use is a significant global public health concern in the contemporary healthcare system with alarming trends, associated with a significant burden worldwide. Based on a report in 2023 by the United Nations Office on Drugs and Crime, there was a 23 percent rise observed over the span of a decade from 2011 to 2021 in the global population aged 15 to 64 reporting drug use within the preceding 12 months, although part of this increase was due to population growth [1]. Additionally, substance use has been linked to a substantial direct and indirect mortality rate, estimated to be approximately half a million deaths in 2019, more than doubling the reported figures from three decades ago. The use of different drugs also comes with various physical and psychological complications and a notable disability‐adjusted life years burden exceeding 30 million years [1]. Studies have revealed that substance use is associated with an increased risk of cardiovascular and cerebrovascular diseases, acute and chronic respiratory system disorders, gastrointestinal tract injuries, infectious diseases, and various types of cancers [2, 3, 4, 5]. Furthermore, substance use may contribute to the onset or exacerbation of a range of mental health problems, including mood disorders, anxiety disorders, and psychotic disorders [6, 7, 8]. Beyond health concerns, it can lead to social and interpersonal problems, such as family conflicts, interpersonal and domestic violence, and crime [9, 10]. A report from the National Drug Intelligence Center estimated that over $193 billion was imposed on society within a single year in the United States of America due to the direct and indirect negative impacts of substance use on health, productivity, and criminal activity [11].

Therefore, substance use stands as a crucial, high‐burden problem demanding further attention and comprehensive intervention strategies. This problem holds particular significance among youth, who constitute the demographic group with the highest rate of substance use, primarily due to the critical developmental periods of adolescence and young adulthood [1, 12]. These periods are characterized by heightened susceptibility to peer influence, experimentation, impulsivity, and novelty‐seeking and risk‐taking behaviors, including substance use, as evidenced by studies indicating that a majority of individuals have their first encounter with substances during this time [13, 14, 15, 16]. Besides, early initiation of substance use during youth can have profound and long‐lasting effects on their physical, psychological, cognitive, and social development [13]. Substance use during this formative period is also associated with a higher likelihood of developing substance use disorders later in life [17]. Moreover, societal and cultural factors, such as shifting social norms, media influence, and economic disparities, can further contribute to the vulnerability of youth to substance use [18].

Given the significant impact of substance use among youth, it is imperative to conduct comprehensive assessments of its current status, usage rates, and associated factors. Currently, several studies have explored aspects of substance use among the high school student population in Iran at the level of cities and provinces. These localized studies provide valuable insights into substance use patterns among Iranian youth, reporting high rates of use [19, 20, 21, 22, 23]. However, a nationwide survey is still lacking, which could further facilitate the development of targeted prevention and intervention strategies through regulatory policies. In this study, we aimed to conduct a nationwide investigation to assess the prevalence and pattern of consumption of various substances among Iranian high school students. Additionally, our objective was to identify the factors associated with the levels of consumption to target high‐risk groups. By identifying these high‐risk groups, more tailored programs can be implemented.

## 2 Materials and Methods

This study employed a cross‐sectional survey design to investigate the prevalence of substance use among high school students across the country during the academic year 2023–2024. The proposal for the study has been approved by the Biomedical Research Ethics Committee of the Faculty of Psychology and Educational Sciences at Shiraz University (IR.US.PSYEDU.REC.1402.104). This study was conducted and reported in accordance with the Strengthening the Reporting of Observational Studies in Epidemiology (STROBE) guidelines (Supporting Information S1: Material 1). After approval of the proposal, we executed the study adhering to the latest version of the Declaration of Helsinki in 2013. Participation in the study was voluntary and upon distribution of the questionnaires, the purpose of the study was explained to the participants. They were also assured of the anonymity and confidentiality of their responses.

### 2.1 Study Participants Recruitment

A convenience sampling technique was utilized in this study for participant recruitment. An online questionnaire was hosted on Google Docs, and its link was disseminated across various educational groups and channels on two widely used instant messaging applications in Iran: WhatsApp and Telegram, which are popular among high school students. Notably, these included channels associated with publications in Iran focused on creating educational content for high school students, for example, those who are preparing for university entrance exams.

The eligible study participants were high school students from all high school grades who were willing to participate in this study. Besides, in this study, only students of the Theoretical branches are included. These students could be from any field of study in the Theoretical branches, including Experimental Sciences, Mathematics & Physics, Literature & Humanity, and Islamic Sciences & Knowledge. Students from other educational branches including Technical & Vocational and Skill & Knowledge (Kar‐Danesh) were not included. However, students who had switched their educational branches to Theoretical branches in preceding years were eligible for participation. Those participants who submitted incomplete questionnaires or were enrolled in other school grades, such as elementary and middle school, were also excluded. We imposed no restriction on the gender, type of school, residential area, or ethnicity, ensuring a diverse representation across different demographics. Naturally, the age of high schoolers in Iran ranges between 15 and 18 years old. However, there are some students who fall behind academically. This was anticipated and planned for as we added an additional age group of “> 18” to the questionnaire.

### 2.2 Data Collection

The data collection process was conducted through the distribution of an online questionnaire comprising two sections. The first section of the questionnaire focused on gathering a comprehensive range of demographic characteristics. Participants were asked to provide detailed information including their age (in years), gender (male or female), marital status (married or nonmarried), school grade (10th, 11th, or 12th grade), province of residence (selected from a complete list of Iran's 31 provinces), residential area (urban or rural), school type (public, nonpublic, and National Organization for Development of Exceptional Talents, NODET, schools), grade point average (ranging from 0 to 20), parental marital status (married or single), and educational level of each parent separately (illiterate, elementary or middle school education, high school education, or academic education). Despite NODET schools being classified as public schools, the distinctive characteristics and specialized educational focus of NODET schools justify its separate consideration in studies examining substance use patterns among students. Therefore, we categorized NODET schools as a distinct class to explore their potential influence on substance use behaviors among the students.

In the second section of the questionnaire, participants were presented with a thorough list of inquiries regarding their history of substance use, encompassing both lifetime use and recent consumption within the past 12 months, which we referred to as recent substance use thereafter. This section closely resembled the structure of questionnaires utilized in prior studies conducted in Iran on substance use [22]. However, we took measures to enhance the comprehensiveness of our data collection process and minimize underreporting by incorporating additional names of various substances in the questionnaire. These included not only conventional terms but also street names, nicknames and company labels commonly used in Iran. The substances queried in this second section were categorized into three classes: depressants, stimulants, and hallucinogens. Depressants encompassed alcohol, beta‐blockers, benzodiazepines, opiates, and tramadol. Stimulants included cigarettes, electronic cigarettes, hookah, methylphenidate, cocaine, and methamphetamine. Lastly, hallucinogens comprised cannabis, lysergic acid diethylamide (LSD), and ecstasy. In addition, alcohol, cannabis, cigarettes, cocaine, ecstasy, electronic cigarettes, hookah, LSD, methamphetamine, and opium were classified as non‐prescription/recreational substances and beta‐blockers, benzodiazepines, methylphenidate, and tramadol were classified as prescription substances.

The item list was developed after a targeted scoping of Iranian student/youth substance‐use literature and regional policy/surveillance reports published between 2000 and 2024, cited throughout the manuscript. We prioritized substances with documented use among Iranian adolescents or strong policy relevance: cigarettes and hookah; e‐cigarettes/vapes; opiates/opium and tramadol; benzodiazepines; psychostimulants (methylphenidate, methamphetamine); and hallucinogenic drugs (ecstasy/MDMA, LSD, cocaine). To minimize under‐reporting, each item included formal names and common Persian street/brand terms. E‐cigarettes were retained despite the national sales ban to capture off‐market access among youth. It should be noted that beta‐blockers were included because of documented student self‐medication for performance anxiety in the region; while not classical depressants, they were grouped with depressant/anxiolytic agents for analytic parsimony.

### 2.3 Statistical Analyses

We performed data management and analyses using Stata software (StataCorp LLC, TX, USA) version 17 and R version 4.5.1. *χ* ^2^ test was used for univariable analyses to assess the association between various categorical variables and ever or recent use of substances. Additionally, independent samples t‐test and one‐way analysis of variance (ANOVA) were employed to explore the relationship between these variables and the number of different substances ever or recently used by individuals. These two continuous variables were the outcome variables in the multivariable analysis models of this study. In these multivariable models, using Poisson regression, relevant variables with a *p*‐value of less than 0.3 in the univariate analyses were included, and then, adjusted prevalence ratios (PRs) and their 95% confidence intervals (CI) were estimated. Statistical significance was determined at a *p*‐value less than 0.05. This study follows the recommendations put forward by Assel et al. [24].

### 2.4 Usage of Artificial Intelligence

Large language models (LLMs) were employed only for language editing (grammar and phrasing) of author‐written text. All scientific content, including study design, statistical analyses, interpretation, and conclusions, was produced by the authors without LLM input.

## 3 Results

A total of 9402 individuals, comprised of 5254 (56.9%) males and 3988 (43.1%) females, participated in the current study. The study primarily comprised adolescents, with 7062 individuals (76.1%) falling within this demographic group. Notably, a substantial proportion consisted of individuals aged 17 and 18 years old, with 3074 and 3175 individuals, respectively. The vast majority of participants were not married (97.6%), and the predominant school grade was 12th grade (47.1%). Among the married participants, 75 individuals, comprising 34.3%, were less than 18 years old. Regarding school type, 4638 individuals (50.2%) attended public schools, 1776 individuals (19.2%) attended nonpublic schools, and 2825 individuals (30.6%) attended NODET schools.

Of the study participants, 5715 (60.8%) individuals abstained from using any substances. Of the 3687 who reported ever using at least one substance, 2691 (73.0%) reported recent substance use, constituting 28.6% of the total study cohort. Significant differences were observed across age groups, with older cohorts exhibiting elevated rates of both ever and recent substance use. These differences were also found within other subgroups of the population, including gender, individual and parental marital status, school grade, school type, grade point average, and parental educational levels. A comprehensive overview of the patterns of substance use across various demographic subgroups among the study participants is provided in Table 1.

**Table 1:** Substance use patterns across different demographic subgroups among the study participants.

Variable	Total population *n* (%)	With ever substance use *n* (%: 95% CI)	*p* value	With recent substance use *n* (%: 95% CI)	*p* value
	9402 (100)	3687 (39.2: 38.2–40.2)		2691 (28.6: 27.7–29.5)	
Age (years)					
15	147 (1.6)	33 (22.4: 16.4–29.9)	< 0.001	26 (17.7: 12.3–24.7)	< 0.001
16	666 (7.2)	165 (24.8: 21.6–28.2)		120 (18.0: 15.3–21.1)	
17	3074 (33.1)	1007 (32.8: 31.1–34.4)		718 (23.4: 21.9–24.9)	
18	3175 (34.2)	1331 (41.9: 40.2–43.6)		977 (30.8: 29.2–32.4)	
18 <	2221 (23.9)	1145 (51.6: 49.5–53.6)		846 (38.1: 36.1–40.1)	
Gender					
Male	5254 (56.9)	1523 (38.2: 36.7–39.7)	0.013	1079 (27.1: 25.7–28.5)	0.001
Female	3988 (43.1)	2141 (40.7: 39.4–42.1)		1595 (30.4: 29.1–31.6)	
Marital status					
Nonmarried	9034 (97.6)	3559 (39.4: 38.4–40.4)	< 0.001	2601 (28.8: 27.9–29.7)	0.001
Married	220 (2.4)	121 (55.0: 48.4–61.5)		86 (39.1: 32.9–45.7)	
School type					
Governmental	4638 (50.2)	1813 (39.1: 37.7–40.5)	< 0.001	1305 (28.1: 26.9–29.4)	< 0.001
Nongovernmental	1776 (19.2)	781 (44.0: 41.7–46.3)		581 (32.7: 30.6–34.9)	
NODET	2825 (30.6)	1071 (37.9: 36.1–39.7)		786 (27.8: 26.2–29.5)	
Residential area					
Rural	333 (3.6)	139 (41.7: 36.6–47.1)	0.431	100 (30.0: 25.3–35.2)	0.654
Urban	8936 (96.4)	3538 (39.6: 38.6–40.6)		2582 (28.9: 28.0–29.8)	
Grade point average (out of 20)					
≤ 10	10 (0.1)	4 (40.0: 15.8–70.3)	< 0.001	4 (40.0: 15.8–70.3)	< 0.001
10 < and ≤ 12	67 (0.8)	31 (46.3: 34.8–58.2)		28 (41.8: 30.6–53.8)	
12 < and ≤ 14	169 (1.9)	87 (51.5: 44.0–58.9)		66 (39.1: 32.0–46.6)	
14 < and ≤ 16	461 (5.2)	241 (52.3: 47.7–56.8)		181 (39.3: 34.9–43.8)	
16 < and ≤ 18	1488 (16.7)	756 (50.8: 48.3–53.3)		542 (36.4: 34.0–38.9)	
18 < and ≤ 20	6733 (75.4)	2432 (36.1: 35.0–37.3)		1765 (26.2: 25.2–27.3)	
Parental marital status					
Single	321 (3.5)	176 (54.8: 49.3–60.2)	< 0.001	144 (44.9: 39.5–50.3)	< 0.001
Married	8907 (96.5)	3492 (39.2: 38.2–40.2)		2534 (28.4: 27.5–29.4)	
Paternal educational level					
Illiterate	132 (1.4)	66 (50.0: 41.6–58.4)	< 0.001	51 (38.6: 30.7–47.2)	0.002
Elementary school education	660 (7.1)	295 (44.7: 40.9–48.5)		220 (33.3: 29.8–37.0)	
High school education	3102 (33.6)	1263 (40.7: 39.0–42.5)		914 (29.5: 27.9–31.1)	
Academic education	5351 (57.9)	2050 (38.3: 37.0–39.6)		1497 (28.0: 26.8–29.2)	
Maternal educational level					
Illiterate	193 (2.1)	88 (45.6: 38.7–52.7)	0.007	61 (31.6: 25.4–38.5)	0.138
Elementary or middle school education	806 (8.7)	360 (44.7: 41.3–48.1)		258 (32.0: 28.9–35.3)	
High school education	3258 (35.2)	1273 (39.1: 37.4–40.8)		915 (28.1: 26.6–29.6)	
Academic education	4996 (54.0)	1961 (39.3: 37.9–40.6)		1454 (29.1: 27.9–30.4)	

Abbreviations: CI, confidence interval; NODET, National Organization for Development of Exceptional Talents.

**Figure 1:**
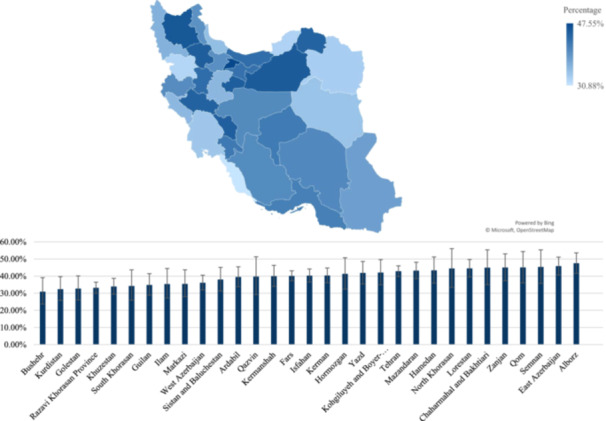
Distribution of ever substance use among the participants from different provinces of Iran.

**Figure 2:**
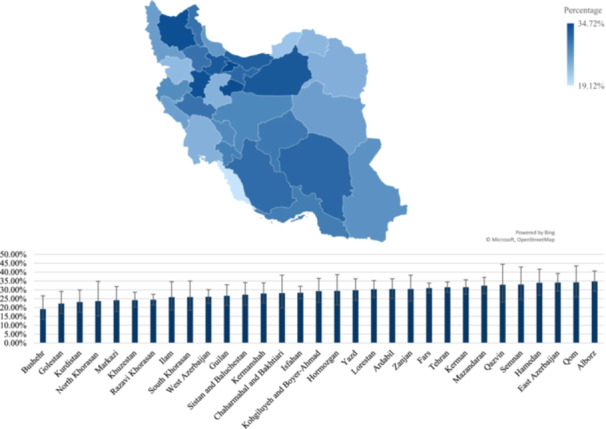
Distribution of recent substance use among the participants from different provinces of Iran.

Figures 1 and 2 demonstrate the prevalence of ever and recent substance use across different provinces of Iran, showing significant variations in substance use prevalence among different regions of Iran. The highest rate of both ever and recent use of substances was in Alborz province with rates of 47.5% (95% CI of 41.6% to 53.6%) and 34.7% (95% CI of 29.2% to 40.6%). Conversely, Bushehr province exhibited the lowest rates, with rates of 30.9% (95% CI: 23.7% to 39.1%) for ever use and 19.1% (95% CI: 13.4% to 26.6%) for recent use.

**Figure 3:**
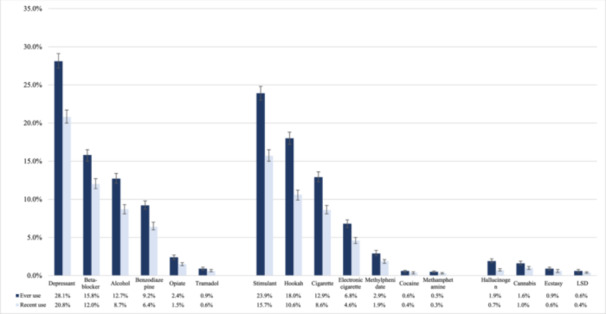
Ever and recent use of various substances by the study participants.

Depressant substances emerged as the most commonly used category among participants, both in terms of ever use and recent use, with 2646 (28.1%) and 1,958 (20.8%) participants, respectively. Within the depressants category, beta‐blockers exhibited the highest ever use rate at 15.8%, followed by alcohol and benzodiazepines at 12.7% and 9.2%, respectively. Stimulant substances showed notable ever use rates at 23.9%, with individual substances ranging from 0.5% for methamphetamines to 18.0% for hookah, while recent use rates were generally lower. Hallucinogens displayed lower prevalence compared to depressants and stimulants, with 183 participants (1.9%) reporting ever use and 66 participants (0.7%) reporting recent use (Figure 3). Additionally, 147 (1.6%) and 54 (0.6%) participants reported ever and recently using all three categories, respectively.

**Figure 4:**
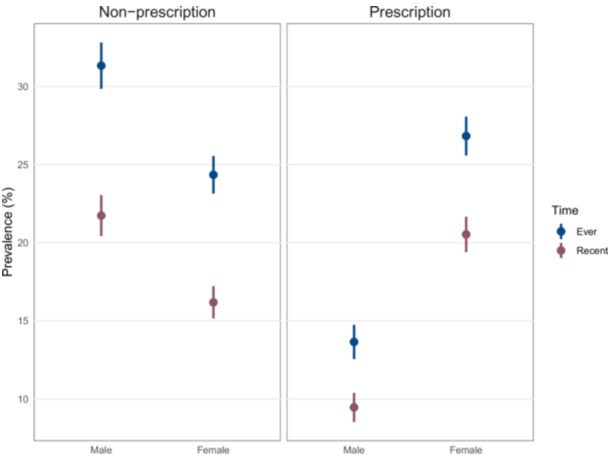
Prevalence of prescription versus non‐prescription/recreational drug use stratified by gender and chronology (recent and ever).

**Figure 5:**
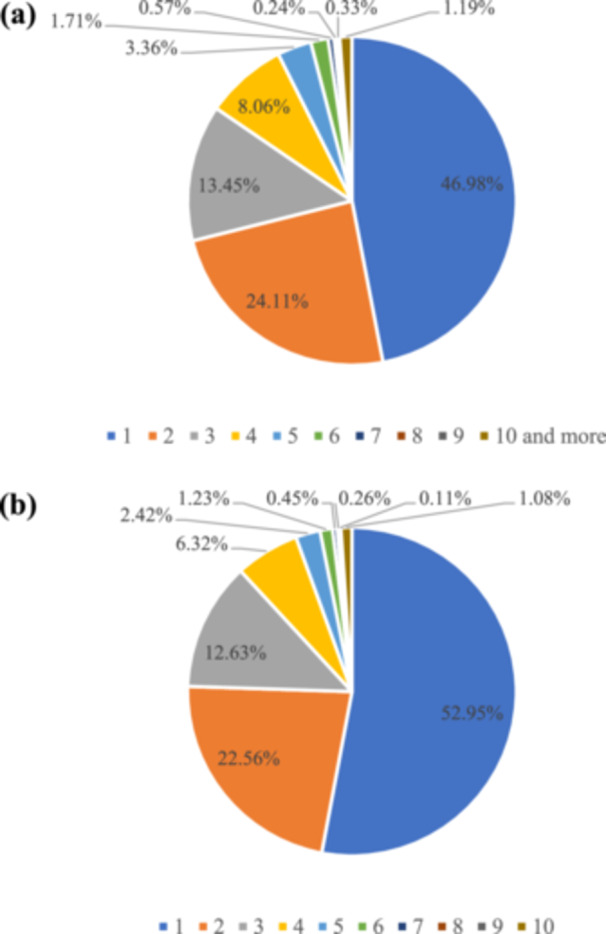
Number of different types of substances (a) ever and (b) recently used by the study participants.

Female participants reported higher use of prescription drugs, whereas male participants showed greater use of non‐prescription/recreational substances. This pattern was evident for both recent and lifetime use (Figure 4). Among the participants who had ever used substance, nearly half of them, 1732 individuals (47.0%), reported ever using only a single substance, with a gradual decrease in percentage as the number of different substances used by the participants increased. A minority of participants reported using ten or more substances, constituting 44 individuals (1.2%) of the total population. In terms of recent substance use, 1,425 individuals (52.9%) reported using only one type of substance within the last year, with a similar pattern of decreasing percentages as the number of different substances used by the participants increased (Figure 5). Supporting Information S2: Material 2 demonstrates a correlation heatmap demonstrating the pairwise co‐abuse of substances with each other. According to Table 2, Ardabil and Alborz exhibit the highest prevalence of recent and ever use, respectively, among males, whereas their prevalence for the other metric is near the median among provinces. In contrast, Semnan has one of the highest rates of both recent and ever use among females.

**Table 2:** The prevalenikug use based on gender and province.

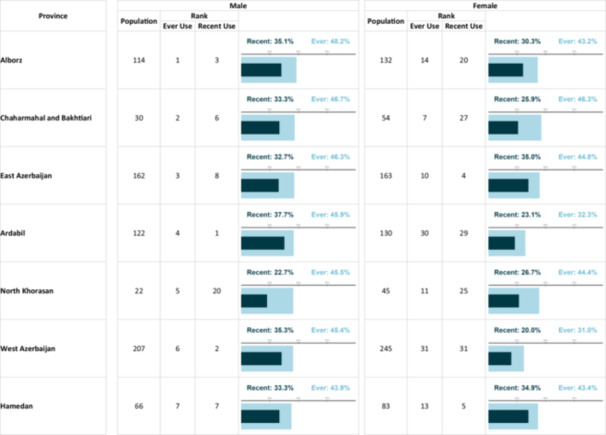
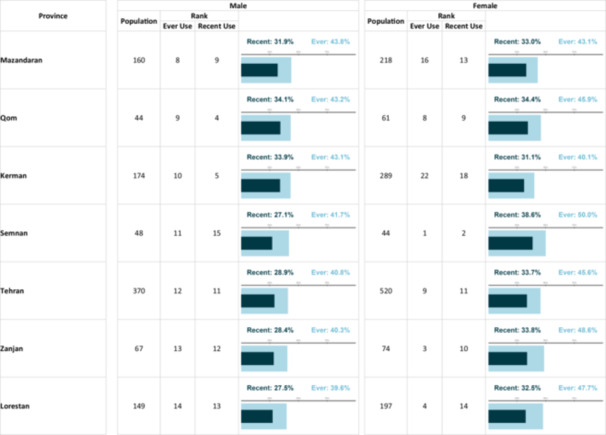
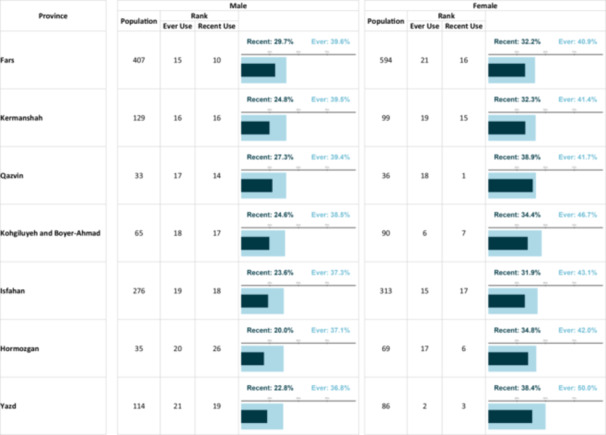
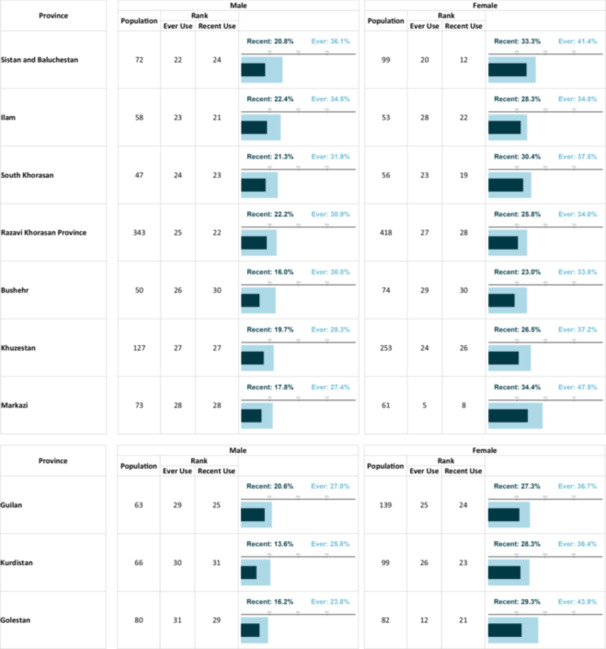

**Figure 6:**
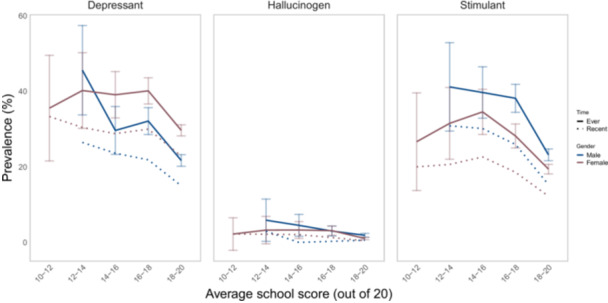
Prevalence of different drug types across average school score (range: 0–20).

Table 3 presents a comprehensive overview of the factors associated with a higher number of different substances ever and recently used by the study participants. The analyses revealed significant associations between various variables and substance use behaviors. Older age, being married, and attendance at nonpublic and NODET schools corresponded to elevated adjusted PRs for the use of a higher number of different substances, both in terms of ever and recent use. On the other hand, individuals with married parents, higher parental educational attainment, and those achieving higher grade point average at school (above 16 compared to 16 and below) exhibited significantly lower numbers of different used substances. Moreover, residing in urban regions and being female were only associated with a decreased number of different substances ever used. Figure 6 demonstrates the prevalence of use across substance type and average school score (range: 0–20), indicating the significantly lower prevalence of hallucinogens among Iranian youths.

**Table 3:** Factors associated with the quantity of different substances ever and recently used by the study participants.

Variable	Ever substance use adjusted prevalence ratio (95% CI)	*p* value	Recent substance use adjusted prevalence ratio (95% CI)	*p* value
Age (Ref: ≤ 18 years)				
18 >	1.56 (1.48–1.64)	< 0.001	1.48 (1.39–1.57)	< 0.001
Gender (Ref: Male)				
Female	0.93 (0.89–0.97)	0.002	0.98 (0.93–1.04)	0.472
Marital status (Ref: Nonmarried)				
Married	1.62 (1.44–1.82)	< 0.001	1.58 (1.37–1.82)	< 0.001
School type (Ref: Governmental)				
Nongovernmental	1.25 (1.17–1.32)	< 0.001	1.29 (1.20–1.39)	< 0.001
NODET	1.18 (1.12–1.25)	< 0.001	1.25 (1.17–1.34)	< 0.001
Residential area (Ref: Rural)				
Urban	0.87 (0.77–0.97)	0.016	0.88 (0.76–1.01)	0.074
Grade point average (Ref: ≤ 16, out of 20)				
16 >	0.74 (0.68–0.79)	< 0.001	0.71 (0.65–0.78)	< 0.001
Parental marital status (Ref: Single)				
Married	0.52 (0.48–0.57)	< 0.001	0.48 (0.43–0.54)	< 0.001
Parental educational level (Ref: Both less than diploma)				
At least one parent with high school diploma	0.88 (0.79–0.98)	0.020	0.82 (0.72–0.93)	0.002
At least one parent with academic education	0.93 (0.84–1.03)	0.169	0.87 (0.77–0.99)	0.034

Abbreviations: CI, confidence interval; NODET, National Organization for Development of Exceptional Talents.

## 4 Discussion

Various studies have been conducted in Iran so far on the prevalence of substance use among different participant populations. The prevalence of substance use among youth in our study was substantial, with 39.2% of participants reporting ever using at least one substance. Moreover, recent substance use was reported by 28.6% of the total cohort. These findings highlight the importance of implementing additional preventive measures to address substance use behaviors among adolescents. It's worth noting that one contributing factor to these high rates is the thoroughness of our questionnaire, which included a comprehensive list of substances compared to several previous studies in Iran [25, 26]. For instance, A national study on substance use prevalence among 130,570 Iranian adults aged 35 and older found that 21.8% reported ever smoking cigarettes and 9% reported alcohol use, results that were not notably lower than ours [26]. However, this study only focused on certain drugs such as opioids, heroin, cocaine, cannabis, and hallucinogens, excluding nicotine products and alcohol when calculating drug use prevalence, and reported a rate of 11.9%, limiting direct comparison of our study with substance use rates among Iranian adults. Nevertheless, the prevalence of substance use in our study aligns with findings from local Iranian high schools where a wide range of substances was assessed, similar to our study [21, 22, 23].

Understanding the pivotal role of parents in influencing their offspring's substance use is paramount. In our study, we investigated two crucial aspects of parental influence and incorporated them into the multivariable analyses. These two variables, parental marital status and parental educational level were both found to be factors significantly associated with the number of different substances ever or recently used by the participants. Specifically, our study revealed that married parental status and higher levels of parental education were associated with a lower number of different substances used by the participants. These findings are consistent with prior research that has consistently demonstrated a negative association between parental marital status and education, and youth substance use across diverse cultural contexts [27, 28, 29, 30, 31]. In a large‐scale study involving 14,268 high school seniors across the United States of America, Small et al. assessed the relationship between various factors and adolescent substance ever abuse [31]. The results indicated that residing with a single mother or without parents was associated with higher odds of substance use compared to those living in a two‐parent household. Additionally, both higher education levels of fathers and mothers were significantly associated with reduced odds of their adolescent children ever using substances.

Parental educational level and marital status thus emerge as significant determinants of adolescent substance use behaviors, reflecting the combined influence of socioeconomic status and parental modeling on youth behaviors [32, 33]. Parents with higher levels of education often possess greater awareness and are more knowledgeable about the risks associated with substance use and may employ more effective parenting strategies to deter their children from engaging in such behaviors. Besides, two‐parent households often provide more parental supervision, communication, and support, creating a nurturing environment that discourages substance use [32]. Moreover, higher parental educational attainment correlates with greater socioeconomic resources, stability, and supportive social networks, which may reduce the likelihood of adolescents resorting to substance use as a coping mechanism [34, 35]. These findings emphasize the importance of targeted interventions aimed at enhancing parental education, strengthening family cohesion, and promoting positive parenting practices to mitigate the prevalence of substance abuse among youth. Additionally, there should also be interventions focusing on the youth not only to have an early detection of substance use among offspring of divorced or widowed families and those with lower familial educational backgrounds but also to improve their awareness regarding the risks associated with substance use and enhancing coping mechanisms [35].

Another important finding of our study was the significant association observed between the individual marital status and ever and recent substance use with married individuals reported using a greater number of different substances. While the association between marital status and substance abuse among youth in our study may seem contradictory, given the common perception that marriage is associated with stability and responsibility, several underlying factors could elucidate this relationship. One key factor is the interplay between specific personality traits and both substance use and marital status [36]. Research has shown that certain personality traits, such as impulsivity and sensation seeking, are associated with an increased likelihood of engaging in substance use [37, 38]. Having early marriage itself has also been shown to be linked to these personality traits, as individuals who marry at a young age may be more impulsive or sensation‐seeking than those who delay marriage. Moreover, the dynamics within marital relationships can influence substance use behaviors. While marriage can provide social support and stability that may protect against substance abuse, it can also introduce stressors and conflicts that may lead to substance use. Studies have found that married individuals undergo significant changes in their relationships and even, in their personalities over time, especially during the initial years of marriage, which may lead to marital dissatisfaction and discord [39, 40, 41]. These shifts in relationship dynamics, along with the added responsibilities and challenges of marriage, could potentially drive individuals, particularly young and less experienced individuals, to cope with stress through substance use [42, 43]. Another plausible explanation could be the sudden acquisition of unsupervised autonomy experienced by young individuals following marriage, which may prompt them to engage in substance use as a means of asserting their newfound independence.

Additionally, we found that participants residing in rural areas have used significantly higher numbers of different substances compared to those participants in urban areas. This finding aligns with several prior research assessing substance use patterns and associated factors, although there are also inconsistent findings [44, 45, 46, 47]. In a cross‐sectional study on high school students from 25 schools across different residential zones of Ethiopia, 3227 participants were selected utilizing a multistage sampling approach [44]. The study reported that rural residents exhibited 2.25 times higher odds of drug usage compared to their urban counterparts. This disparity in substance use is present not only between rural and urban settings but also within different subgroups of these areas, further underscoring the influential role of residential environments in shaping substance use behavior [45]. Expanding on this observation, a study investigated substance use among 18,767 youths attending schools in different rural settings, including residing on farms, in country regions other than farms, or in towns, across seven states in the United States of America [48]. The analyses revealed that high school students living on farms demonstrated higher prevalence rates of various substances, including depressants, stimulants, and hallucinogens, compared to those residing in towns. The heightened use of substances among youth residing in rural areas may be attributed to a multitude of factors, including limited access to healthcare, mental health, and counseling services, socioeconomic disparities, inadequate parental supervision and awareness, cultural norms promoting substance use, and social acceptance toward particular substances, occupational hazards, and a lack of recreational activities.

We observed a difference of substance abuse between genders favoring higher ever and recent use in females in crude univariate analysis. However, gender differences flipped after adjustment, a probable implication that females in our sample were more represented in higher‐risk strata. Adjustment removed that effect, revealing lower lifetime use in females but parity in recent use. A potential justification for the observed effects is that males may initiate earlier and accumulate more ever use while females may initiate later, so recent use levels converge despite lower lifetime exposure in females. In addition, equal recent use alongside lower ever use for females could mean shorter use histories or higher persistence among active users. However, the current study fails to prove these speculations methodologically as it lacks duration data. Although the overall adjusted pattern shows parity in recent use, provinces likely deviate. We observed provinces where female recent use approximates or exceeds males. This indicates that policy‐makers should adopt province‐specific strategies and avoid generalizing the overall findings. Policy‐makers should be advised that gender convergence in active use. Prevention should prioritize delayed initiation (especially in males), while treatment, counseling, and harm‐reduction must address both genders equally. Also, policy‐makers should take into consideration that females tend to use prescription substances while males tend to use recreational substances.

Our investigation also uncovered a significant difference in the usage of various substances among participants attending different types of schools, including public, nonpublic, and NODET schools. Interestingly, the role of school type on substance use has not been widely studied in the current literature, making our findings particularly noteworthy. A similar study conducted in Kerman, Iran, focusing on 650 high school students, reported notable differences in substance use rates based on school type [49]. Specifically, the study found that the rates of cigarette and marijuana use were significantly higher among students attending nonpublic schools compared to those in public schools. In our study, unlike this other Iranian study, we categorized schools into three classes: public, nonpublic, and NODET. While NODET schools are technically public, we considered them as a separate class due to their unique educational framework aimed at nurturing gifted and talented students selected through highly competitive entrance exams. Our findings validated our decision to categorize school types in this manner, revealing distinct substance use patterns in NODET schools compared to other public schools, evident in the rate of both overall and recent usage. Discrepancies in school environments, peer influences, and academic stress levels appear to be the primary underlying factors contributing to this heightened rate of substance use [49, 50]. While adolescents attending nonpublic and NODET schools are often from families with higher socioeconomic status and parental educational levels, factors typically associated with lower rates of substance use, the academic rigor associated with nonpublic and NODET schools may expose students to increased peer pressure and social influences conducive to substance experimentation, compounded by the higher affordability of these substances.

The current study was the first nationwide study that explored substance use patterns and their associated factors among high school students. To mitigate the risk of underreporting, we meticulously compiled a comprehensive list of various substances alongside their alternative names in our questionnaire. Another notable strength of our study was the utilization of a multivariable model with a continuous outcome measure, assessing the number of various substances ever and recently used by participants, rather than employing a binary outcome of whether the participants have experience of substance use or not. This approach allowed for a more nuanced analysis of factors associated with substance use behavior among youths. Unlike a binary outcome, which treats all substance users equally regardless of the number of substances used, our methodology provided a more accurate representation of substance use patterns, enabling a deeper understanding of the factors influencing such behaviors. Moreover, another notable aspect of our study was our approach to the classification of types of schools. While previous studies in Iran categorized schools as either public or nonpublic, we introduced a novel distinction by separating NODET schools from other public schools. This innovative classification strategy led to the discovery of a noteworthy observation.

### 4.1 Limitations

Our study also had some limitations. Firstly, and most importantly, we utilized a convenience sampling technique, disseminating the questionnaire through online platforms accessible to high school students. Due to non‐probability sampling, this approach may have introduced selection bias and limited generalizability, as it predominantly captured responses from students who are more active on social media, potentially excluding individuals with limited internet access or those less inclined to participate in online surveys. Besides, the data collection relied on self‐reported responses, which are susceptible to recall bias and social desirability bias. Third, our study had a cross‐sectional design, which restricts use to establish causal relationships between variables. Longitudinal studies are, therefore, needed to elucidate the temporal sequence of events and explore the trajectories of substance use behaviors over time among high school students. The questionnaire's target audience was adolescents and young adults who were in the process of preparing for the National Entrance Exam (Konkur). This highly demanding evaluation exam requires years of hard work and studying. Therefore, the researchers anticipated that an extensive questionnaire would be beyond the attention span of the target audience. Consequently, the questionnaire was designed to be simple, easy to read, and to the point. Inherently, such a concise questionnaire would be unable to capture every plausible variable. This is to say that the authors confirm that some potentially relevant variables, such as emotional state, socioeconomic status, and geographical access to substances, are missing from the study. We did not include separate items for some novel psychoactive substances (e.g., synthetic cannabinoids/nitazenes) due to limited Iran‐specific evidence in high‐school populations and measurement validity concerns.

## 5 Conclusions

The current study investigated substance use patterns among a large cohort of participants in Iran, shedding light on the prevalence, correlates, and implications of substance use in this population. A notable percentage of high school students was found in this study to have the experience of ever and recent substance use, highlighting concerning figures in this population. Additionally, our findings revealed significant associations between substance use and several demographic factors, including participants' age and gender, the type of schools attended by the participants, and the characteristics of their families, underscoring the complex interplay of individual, familial, and socioenvironmental factors in shaping adolescent substance use behavior. Understanding these complexities could help establish more effective prevention strategies with a particular focus on youths with these associated factors. To attain a more comprehensive and representative understanding, future studies employing robust sampling methodologies, such as in‐school data collection, are warranted. By shedding light on the current status of substance use in the region, our findings contribute to the ongoing efforts to expand knowledge and guide evidence‐based interventions aimed at mitigating the negative consequences of substance use among Iranian youths.

## Author Contributions

F.H., H.H., and E.T. were responsible for conceptualizing and designing the study. Data collection was carried out by F.H. and A.S. H.H., A.S., and E.T. were involved in data cleaning and statistical analyses. F.H. and A.S. contributed to drafting the manuscript, while H.H. and E.T. participated in revising the draft. This study is a part of the thesis by the author, Farshad Hadianfard, for obtaining a completion certificate for his alternative conscription service. All authors have read and approved the final version of the manuscript. Dr. Alireza Sadeghi had full access to all of the data in this study and takes complete responsibility for the integrity of the data and the accuracy of the data analysis.

## Conflicts of Interest

The authors declare no conflicts of interest.

## Transparency Statement

The lead author, Habib Hadianfard, affirms that this manuscript is an honest, accurate, and transparent account of the study being reported; that no important aspects of the study have been omitted; and that any discrepancies from the study as planned (and, if relevant, registered) have been explained.

## Supporting information

STROBE Statement—Checklist of items that should be included in reports of ***cross‐sectional studies***.

Supplmentary Material.

## Data Availability

The data that support the findings of this study are available on request from the corresponding author, Alireza Sadeghi, at alireza.sadeghi.md@gmail.com. The data are not publicly available due to privacy or ethical restrictions.
