# Supplementary figures and images for: Substance Use Among Iranian Youth: A Nationwide Cross‐Sectional Study on the Prevalence, Pattern, and Its Associated Factors
Source: Health Sci Rep. 2025 Dec 3;8(12):e71604. doi: 10.1002/hsr2.71604 (PMC12675134; doi:10.1002/hsr2.71604)

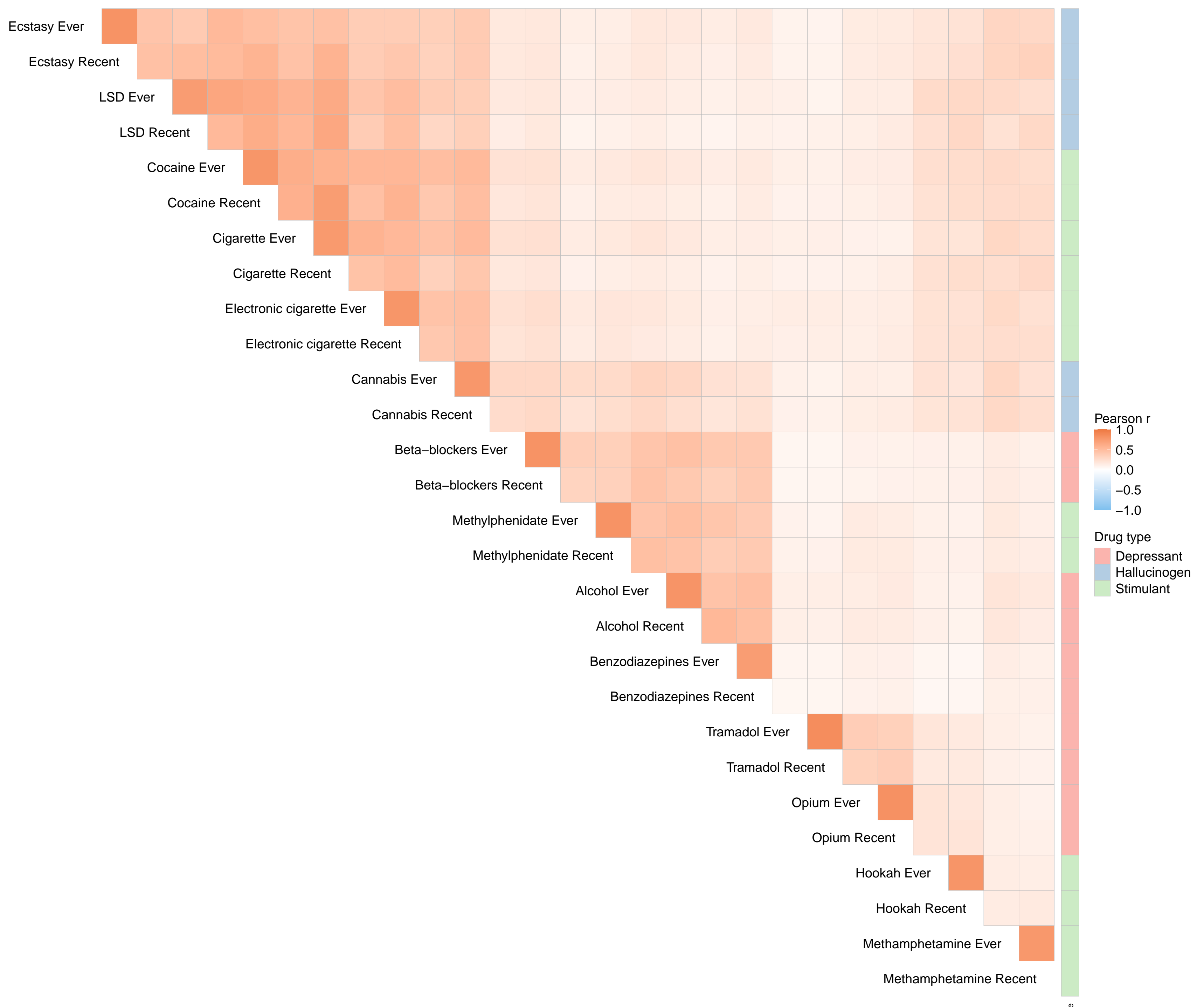

Supplement: Supplementary file 2 — Supplmentary Material. [file HSR2-8-e71604-s001.pdf]
